# Supplementary material for: Human iPSC-derived neural stem cells displaying radial glia signature exhibit long-term safety in mice
Source: Nat Commun. 2024 Nov 1;15:9433. doi: 10.1038/s41467-024-53613-7 (PMC11530573; doi:10.1038/s41467-024-53613-7)
Supplement: Supplementary file 1 — Supplementary Information [file 41467_2024_53613_MOESM1_ESM.pdf]

Supplementary information

- **Supplementary Figures**

Supplementary Figure 1. Expression of NSC markers and predicted TF network in hiPSC-NSCs..

Supplementary Figure 2. Transcription factors upregulated during hiPSC neural differentiation.

Supplementary Figure 3. Analyses of transcription factors downregulated during hiPSC neural commitment and RG markers expressed in hiPSC-NSCs.

Supplementary Figure 4. Validation of ChIP-seq analysis in hiPSCs, hiPSC-NSCs, and hfNSCs.

Supplementary Figure 5. Comparable activation of cell cycle and metabolic pathways in hiPSC-NSCs and hfNSCs.

Supplementary Figure 6. Single-cell RNA-seq analyses in hiPSC-NSCs and hfNSCs.

Supplementary Figure 7. Engrafted hiPSC-NSCs migrate along the rostro-caudal axis and primarily differentiate into glia progenitors.

Supplementary Figure 8. Molecular analyses in SREBP1-deficient cells.

Supplementary Figure 9. Sample gating strategies in flow cytometry analyses.

- **Supplementary Tables**

Supplementary Table 1. Summary of hiPSC clones generated by reprogramming of healthy donor (HD) fibroblasts.

Supplementary Table 2. Upstream regulators identified by Ingenuity Pathway Analysis (IPA) on RNA-seq analysis of hiPSC-NPCs vs. hiPSCs.

Supplementary Table 3. Upstream regulators identified by Ingenuity Pathway Analysis (IPA) on the dataset of genes close to hiPSC- and hiPSC-NSC-specific enhancers

Supplementary Table 4. Upstream regulators identified by Ingenuity Pathway Analysis (IPA) on RNA-seq analysis of hiPSC-NSCs vs. hfNSCs.

Supplementary Table 5. List of gRNAs and primers used for PCR amplification and sequencing of Cas9 on-target sequence. Sequencing results showing the Cas9-nuclease edited sequences (SREBF1 exon 5) in SREBP1-deficient clones.

Supplementary Table 6. List of primers used for qRT-PCR on immunoprecipitated chromatin.

Supplementary Table 7. List of primers and probes used for SYBR Green and TaqMan qRT-PCR.

Supplementary Table 8. List of primary and secondary antibodies with antigen, host species, provider, product number, and working dilutions indicated.

Supplementary figures

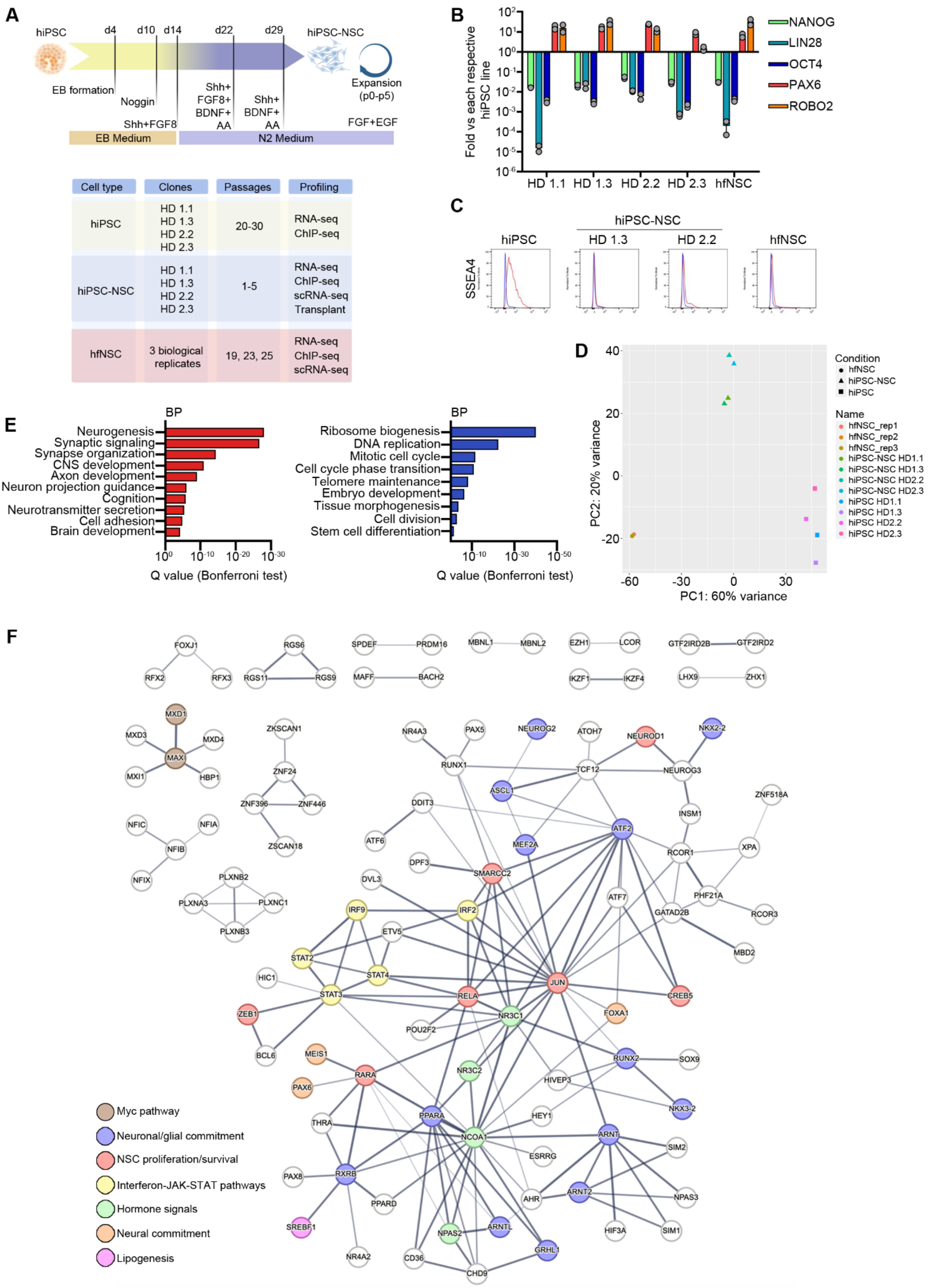

Supplementary Figure 1. Expression of NSC markers and predicted TF network in hiPSC-NSCs. A) Schematic representation of neural differentiation protocol. Timepoints analyzed in this study correspond to: (i) different stages of hiPSC-to-NSC commitment (hiPSCs, day 0; Embryoid bodies, day 4; early and late rosette-like formations, days 10 and 14; hiPSC-NSC maturation, days 22 and 29); (ii) hiPSC-NSC expansion in growth media (passages 0-5). Cell types, clones, passages and -OMICS/functional analyses are reported. Created in BioRender. Garsia, C. (2023) BioRender.com/v79l313. **B)** Bar plot showing the upregulation of NSC markers (*PAX6, ROBO2)* and downregulation of hiPSC markers (*NANOG, LIN28, OCT4*) in hiPSC-NSCs at levels similar to hfNSCs. For each gene, the expression levels in hiPSC-NSCs are reported as fold changes vs. the corresponding parental hiPSC clone, whereas expression levels in hfNSCs are reported as fold changes vs. the mean values in hiPSC clones. Data are expressed as mean ± SEM of n = 2 independent experiments. **C)** Representative FACS plots of SSEA4 (pluripotency marker) expression in hiPSCs, hiPSC-NSCs (clones HD 1.3 and HD 2.2), and hfNSCs. Blue lines, unstained cells; red lines, stained cells. **D)** Principal Component Analysis (PCA) plot of RNA-seq samples comparing hiPSCs vs. hiPSC-NSCs vs. hfNSCs. **E)** Bar plots of biological processes upregulated (red bars) or downregulated (blue bars) in hiPSC-NSCs vs. hiPSCs. **F)** Protein-protein interaction network functional enrichment analysis (STRING) of TFs upregulated in hiPSC-NSCs vs. hiPSCs ($\log_2$ fold change ± 1, adjusted p-value < 0.05). Proteins are colored according to NSC functions defined based on published data. **B-F)** Analyses were performed in hiPSCs clones HD 1.1, HD 1.3, HD 2.2, and HD 2.3 (p20-30); hiPSC-NSC clones HD 1.1 (p3), HD 1.3 (p5), HD 2.2 (p3), and HD 2.3 (p4); hfNSCs: three biological replicates at different passages (p19, p23, p25). Source data are provided as a Source Data file.

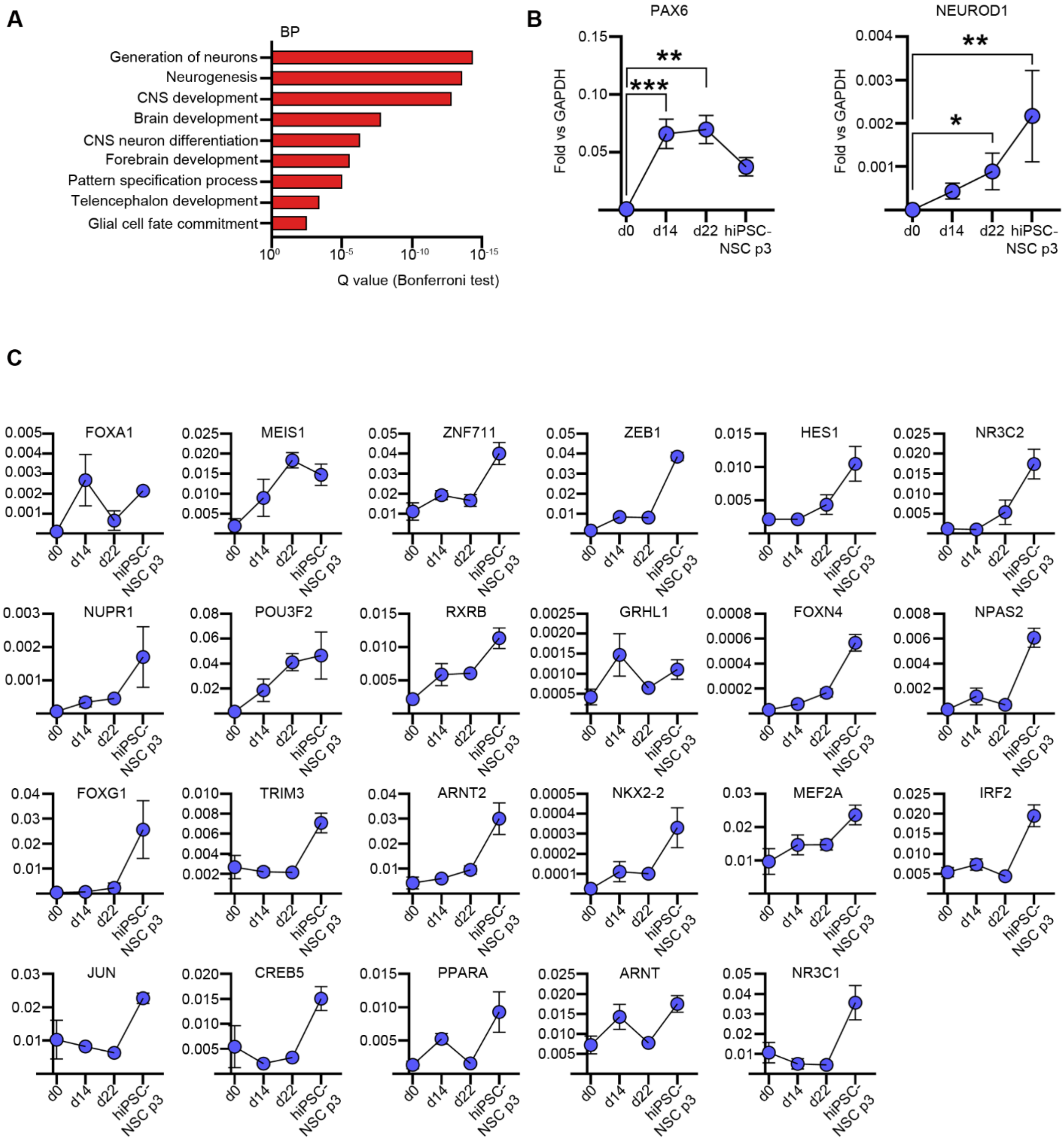

Supplementary Figure 2. Transcription factors upregulated during hiPSC neural differentiation. A) Gene ontology enrichment analysis of transcription factors upregulated in hiPSC-NSCs as compared to parental hiPSCs (log_2 fold change > 1, adjusted p-value < 0.05). Bar plot shows selected biological processes upregulated in hiPSC-NSCs. **B)** qRT-PCR analysis of *PAX6* and *NEUROD1* expression during hiPSC neural differentiation. Expression levels are normalized on the housekeeping gene *GAPDH*. Each dot represents the mean ± SEM of at least n=5 biological replicates/time point. One-way ANOVA followed by Dunn's multiple comparison test: *, $p < 0.05$; **, $p < 0.01$; ***, $p < 0.001$. **C)** Time-course qRT-PCR analysis showing the expression of TFs during hiPSC-to-NSC differentiation. Expression levels are normalized on the housekeeping gene *GAPDH*. Each dot represents the mean ± SEM of at least n=2 biological replicates/time point. **B-C)** Timepoints analyzed: hiPSCs (day 0), rosette-like formations (day 14), hiPSC-NSC maturation (day 22), and hiPSC-NSCs at passage 3. **A-C)** Analyses were performed in hiPSC clones HD 1.1, HD 1.3, HD 2.2, and HD 2.3 (p20-30); hiPSC-NSC clones HD 1.1 (p3), HD 1.3 (p5), HD 2.2 (p3), and HD 2.3 (p4). Source data are provided as a Source Data file.

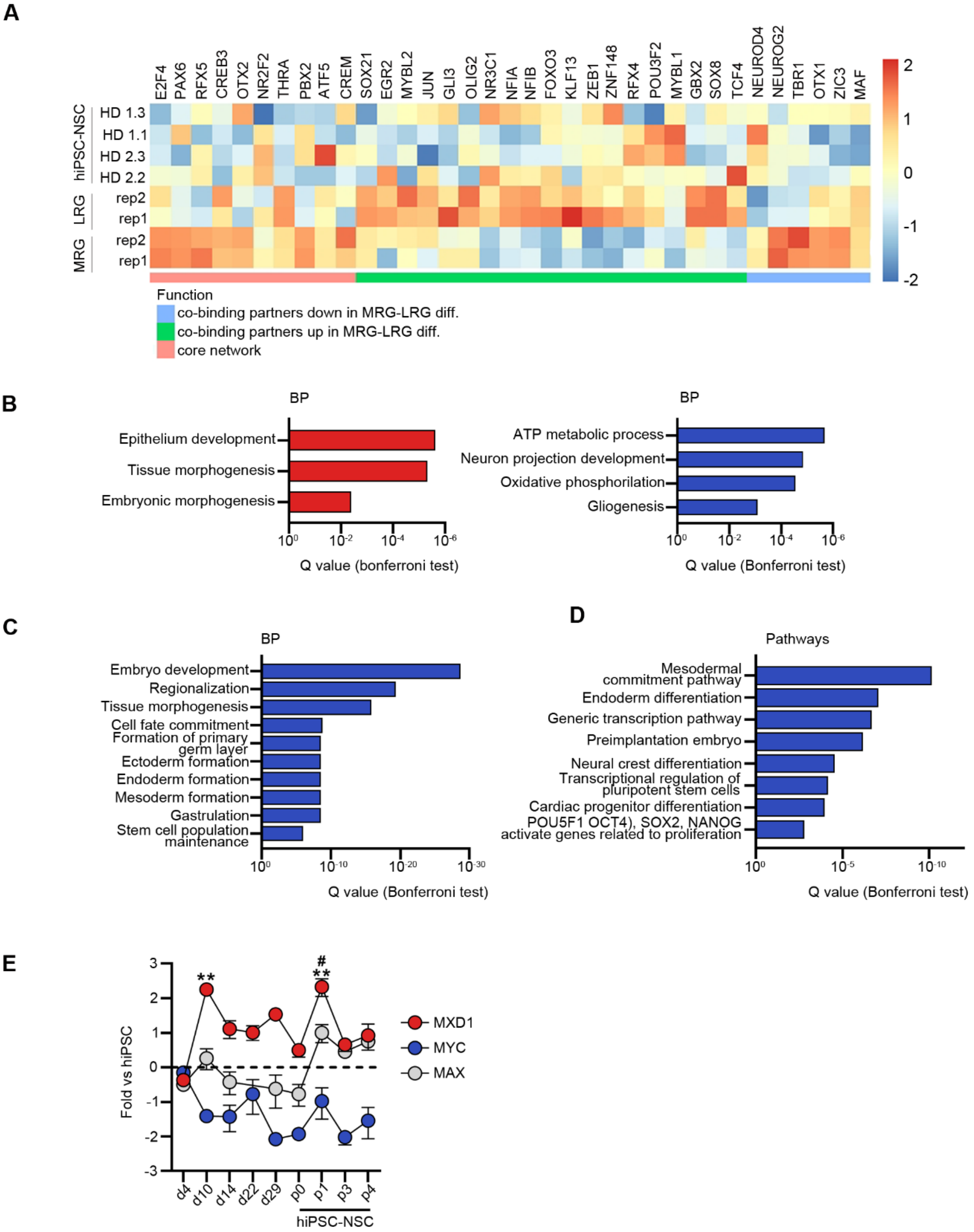

Supplementary Figure 3. Analyses of transcription factors downregulated during hiPSC neural commitment and RG markers expressed in hiPSC-NSCs. A) Heatmap showing the expression levels of core and co-binding TFs regulating ESC neural commitment[77] in hiPSC-NSCs as compared to ESC-derived MRG and LRG. Color scale indicates the average expression levels of these genes in each cell population (blue, low; red, high). **B)** Bar plots showing selected biological processes based on gene ontology enrichment analysis of upregulated (red bars) and downregulated (blue bars) genes in hiPSC-NSCs vs. ESC-derived LRG[77] ($\log_2$ fold change ± 1, adjusted *p*-value < 0.05). **C-D)** Gene ontology enrichment analysis of transcription factors downregulated in hiPSC-NSCs as compared to parental hiPSCs ($\log_2$ fold change > 1, adjusted *p*-value < 0.05). Bar plot shows selected biological processes (C) and pathways (D). **E)** qRT-PCR analysis of *MYC*, *MAX*, and *MXD1* expression during hiPSC-to-hiPSC-NSC transition (timepoints analyzed: Embryoid bodies (day 4), early and late rosette-like formations (days 10 and 14), hiPSC-NSC maturation (days 22 and 29), and hiPSC-NSC at passages 0,1,3 and 4). Expression levels are normalized on the housekeeping gene *GAPDH*.

Each dot represents the mean ± SEM of at least n=3 biological replicates/time point. One-way ANOVA followed by Dunn's multiple comparison test: **, $p < 0.01$ (vs. d4); #, $p < 0.05$ (vs. hiPSC-NSC p0). **A-E)** Analyses were performed in hiPSCs clones [HD 1.1, HD 1.3, HD 2.2, and HD 2.3 (p20-30)] and/or hiPSC-NSC clones [HD 1.1 (p3), HD 1.3 (p5), HD 2.2 (p3), and HD 2.3 (p4)]. Source data are provided as a Source Data file.

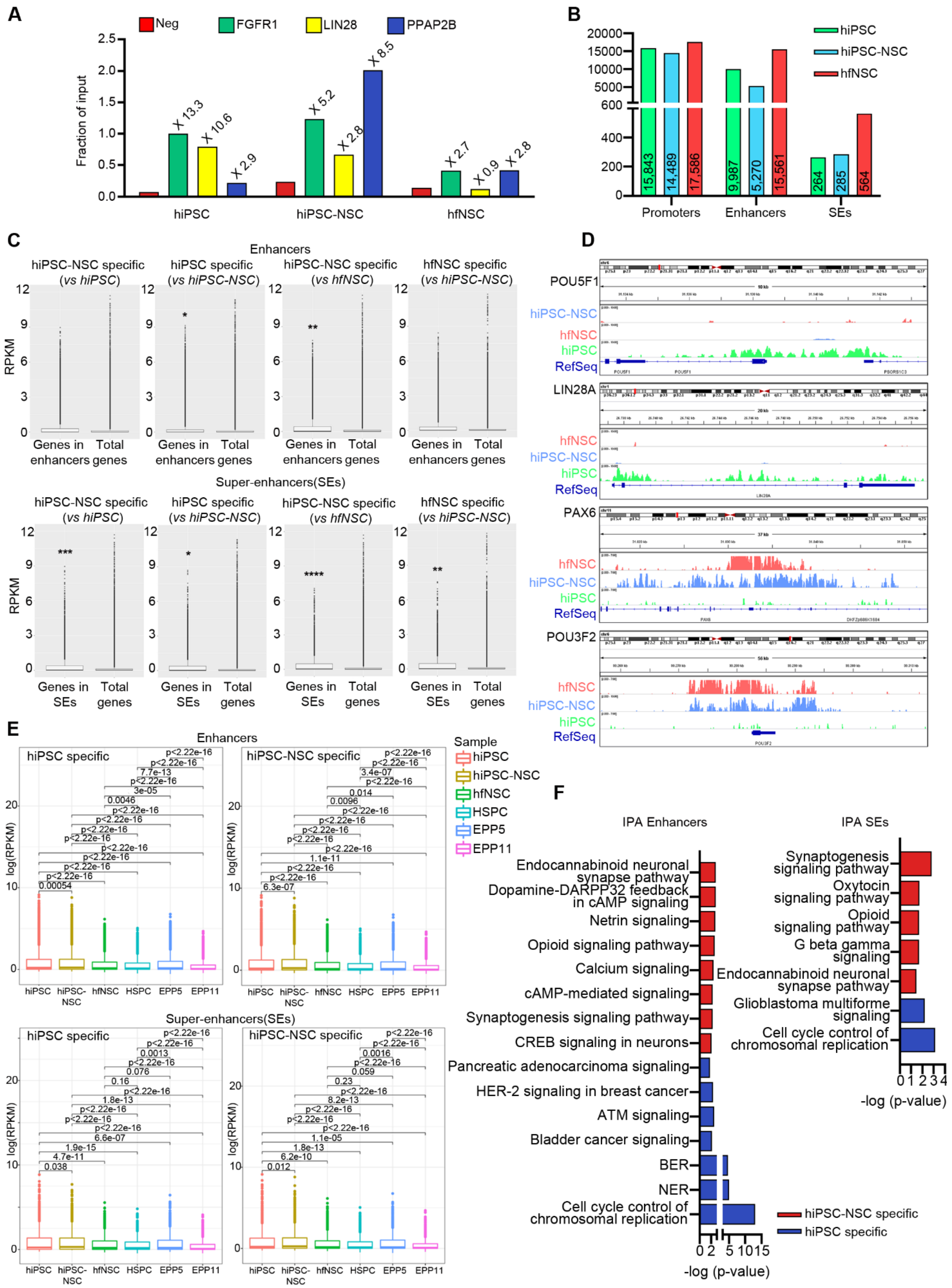

Supplementary Figure 4. Validation of ChIP-seq analysis in hiPSCs, hiPSC-NSCs, and hfNSCs. A) Representative qRT-PCR on H3K27ac[+] immunoprecipitated chromatin for genomic regions containing pluripotent (*LIN28*) and NSC (*PPAP2b*) markers. *FGFR1* serves as positive control in all cell populations while chr13:65364840+65364921 region (82 bp) serves as negative control (Neg). Data are represented as fold

change vs. the corresponding input sample (fraction of input). Enrichment on negative fraction (background) is indicated. **B)** Bar plot showing the number of H3K27ac$^+$ reads corresponding to promoters, enhancers, and SEs identified in each cell population. In each bar are indicated the numbers of H3K27Ac$^+$ reads. **C)** Box plot of expression levels (Reads Per Kilobase Million; RPKM) of genes close to cell-specific enhancers and SEs in comparison with total gene expression levels in each cell population. Welch Two-sample t-test, *, *p-adj* < 0.05; **, *p-adj* < 0.01; ***, *p* < 0.001, ****, p-adj < 0.0001 **D)** Integrative Genomic Viewer **(**IGV) snapshot of H3K27ac$^+$peaks at pluripotent (*POU5F1, LIN28A*) and NSC (*PAX6, POU3F2*) genes in hiPSCs, hiPSC-NSCs, and hfNSCs. Genomic scale and RefSeq gene are indicated. **E)** Box plot of expression levels (logRPKM) of genes close to hiPSC- and hiPSC-NSC-specific enhancers and SEs detected in the comparison of hiPSC and hiPSC-NSC ChIP-seq datasets. To verify the specificity of selected regulatory regions, the expression levels of selected genes were evaluated in published RNA-seq datasets retrieved from human stem/progenitor cells (HSPC) and erythroid progenitor/precursor cells at different stages of maturation (EPP5: day 5, EPP11: day 11)[90]. Pairwise Wilcoxon test was used to determine significant differences in the expression values between different cell types. **F)** Bar plots showing the IPA analysis of up- and down-regulated genes ($\log_2$ fold change ± 1, adjusted *p*-value < 0.05) close to cell-specific enhancers (left plots) and SEs (right plots) in hiPSCs (blue bars) or hiPSC-NSCs (red bars). **A-F)** Analyses were performed in hiPSCs clones HD 1.1, HD 1.3, HD 2.2, and HD 2.3 (p20-30); hiPSC-NSC clones HD 1.1 (p3), HD 1.3 (p5), HD 2.2 (p3), and HD 2.3 (p4); hfNSCs: three biological replicates at different passages (p19, p23, p25). Source data are provided as a Source Data file.

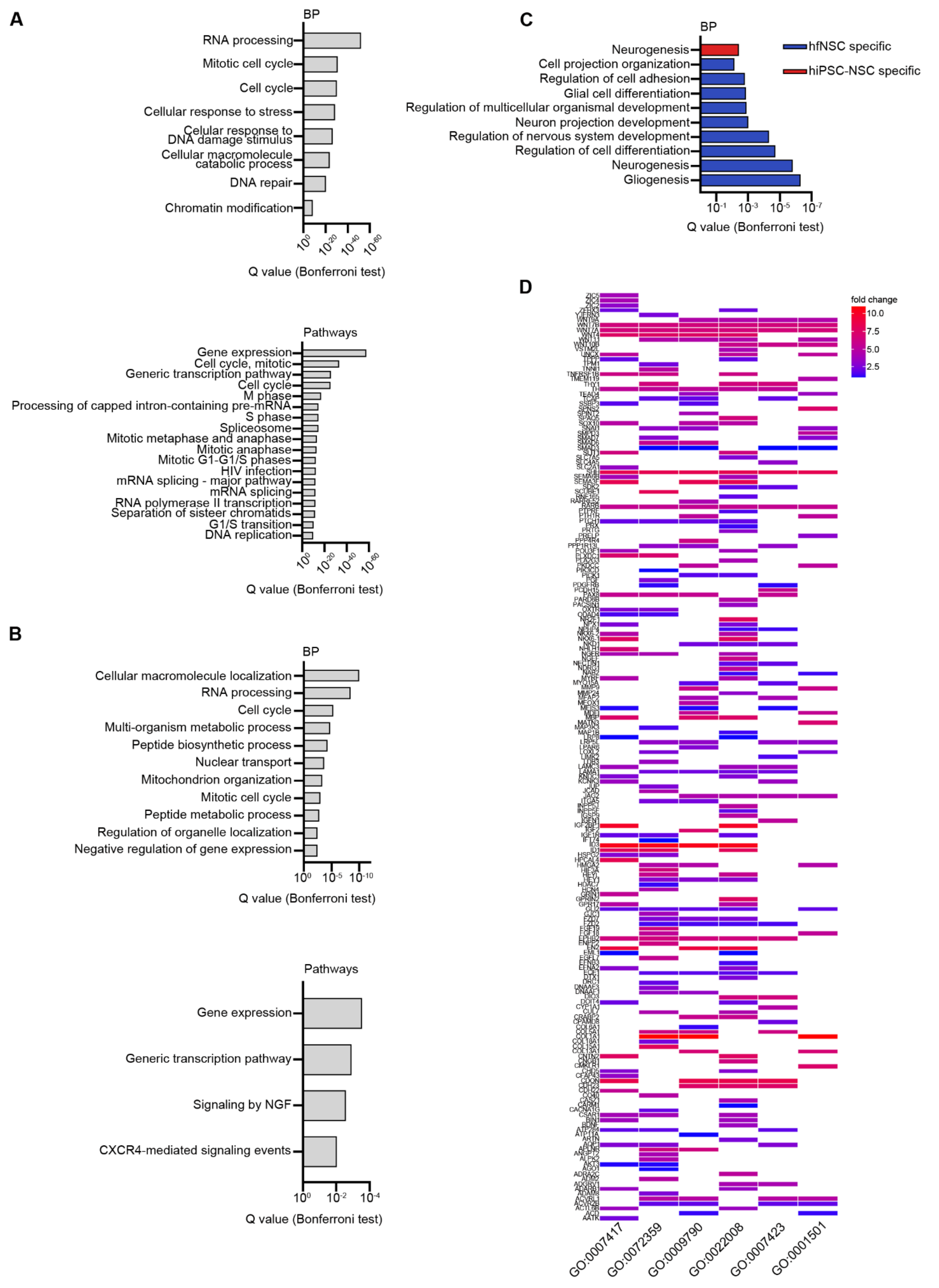

Supplementary Figure 5. Comparable activation of cell cycle and metabolic pathways in hiPSC-NSCs and hfNSCs. A) Gene ontology enrichment analysis of genes not differentially expressed between hiPSC-NSCs and hfNSCs detected in RNA-seq analyses. Bar plots indicate selected biological processes (BP, upper plots) and pathways (lower plots) similarly activated in the two neural populations. **B)** Gene ontology

enrichment analysis of genes close to common enhancers in hiPSC-NSCs and hfNSCs. Bar plots show selected BP (upper plots) and pathways (lower plots) similarly activated in the two neural populations. **C)** Bar plot showing BP associated with genes close to hiPSC-NSC-specific (red bar) and hfNSC-specific (blue bars) SEs. **D)** Heatmap of Cluster Profiler analysis showing genes shared among GO terms associated with neural (GO:0007417, GO:0022008) and non-neural (GO:0072359, GO:0009790, GO:0007423, GO:0001501) biological processes. Fold change enrichment is indicated by the color scale (white, low; red, high). **A-D)** Analyses were performed in hiPSC-NSC clones HD 1.1 (p3), HD 1.3 (p5), HD 2.2 (p3), and HD 2.3 (p4); hfNSCs: three biological replicates at different passages (p19, p23, p25). Source data are provided as a Source Data file.

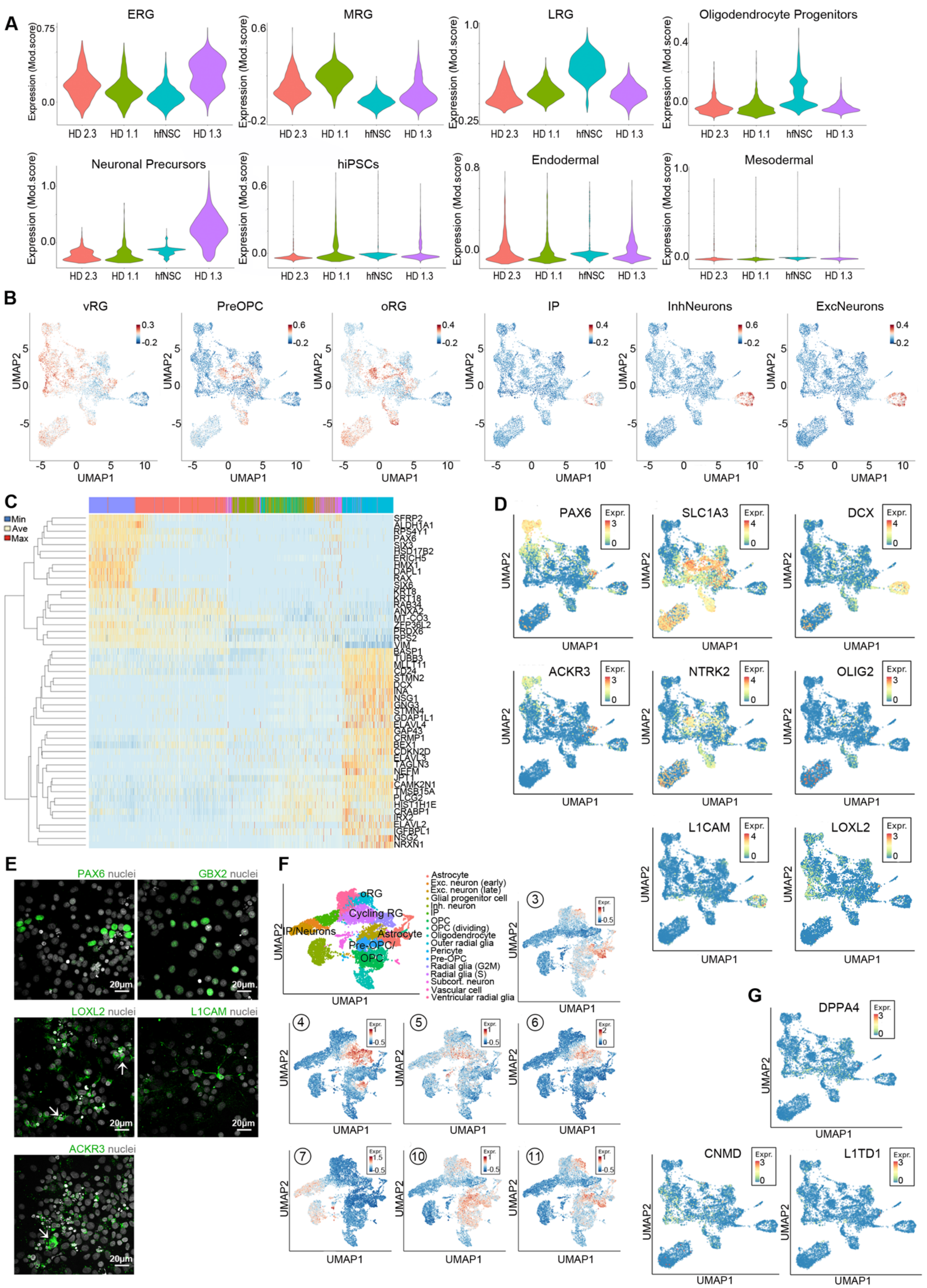

Supplementary Figure 6. Single-cell RNA-seq analyses in hiPSC-NSCs and hfNSCs. A) Violin plots showing the expression levels in hiPSC-NSCs (clones HD 1.1, HD 1.3, and HD 2.3) and hfNSCs of gene signatures of ERG, MRG, LRG, Oligodendrocyte Progenitors, Neuronal Precursors, hiPSCs, Endodermal cells, and Mesodermal cells. **B)** UMAP plot showing the distribution in scRNA-seq samples of cells expressing

markers of cell populations isolated from fetal human brain (ventricular RG, vRG; outer RG, oRG; Pre-OPC; Intermediate Progenitors, IP; inhibitory neurons, InhNeurons; excitatory neurons, ExcNeurons)[118]. Expression is depicted according to the color scale (blue, low; red, high). **C)** Heatmap indicating the expression levels of the top 50 genes along the pseudotime trajectory from Cluster 1 to Cluster 7. Expression is depicted according to the color scale (blue, low; red, high). **D)** UMAP plots showing the distribution of cells expressing membrane-bound markers (*ACKR3*, *NTRK2*, *L1CAM*) in *PAX6*$^+$ early RG, *SLC1A3*$^+$ mature RG, *OLIG2*$^+$ OPC and *DCX*$^+$ neuronal progenitors. *LOXL2* has been identified as a marker of glia progenitors. **E)** Immunofluorescence analyses showing the expression of PAX6, GBX2, LOXL2, L1CAM and ACKR3 proteins in hiPSC-NSCs. Expression is depicted according to the color scale (green, low; red, high). **F)** UMAP plot of top 50 genes that identify hiPSC-NSC/hfNSC-derived mature RG (Clusters 3-6 and 11) and committed progenitors (clusters 7, 10) in scRNA-seq datasets of human fetal brain tissues[118]. Expression is depicted according to the color scale (blue, low; red, high). **G)** UMAP plot showing minimal expression of hiPSC-associated markers (*CNMD, DPPA4*, and *L1TD1*) in scRNA-seq samples. Expression is depicted according to the color scale (green, low; red, high). **A-G)** Analyses were performed in hiPSC-NSC clones HD 1.1 (p2), HD 1.3 (p1), and HD 2.3 (p2); hfNSCs (p19).

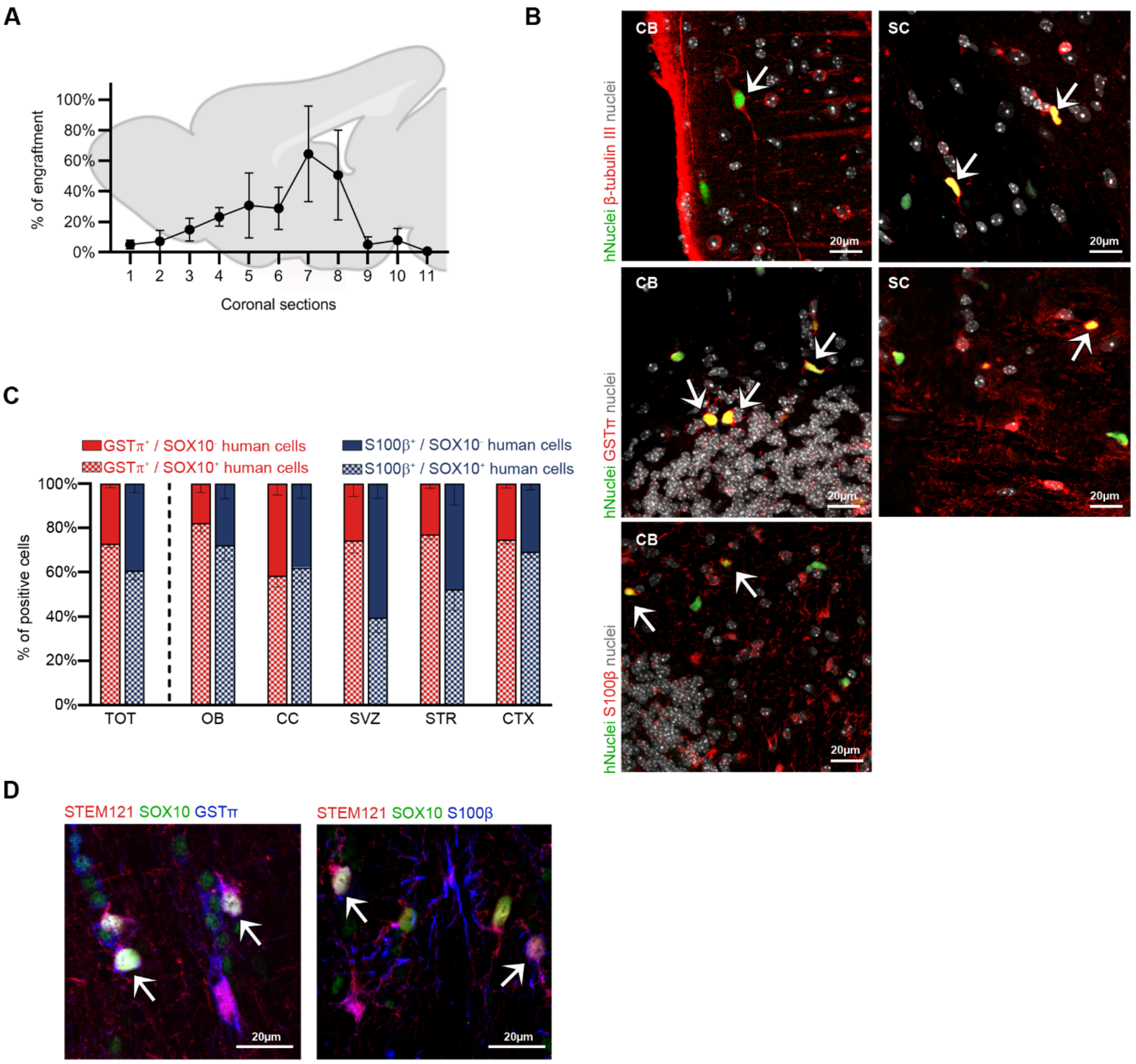

Supplementary Figure 7. Engrafted hiPSC-NSCs migrate along the rostro-caudal axis and primarily differentiate into glia progenitors. A) Graph showing the distribution of engrafted cells based along the rostro-caudal axis, evaluated as percentage of hNuclei$^+$ cells in sequential coronal sections. Data are represented as mean ± SEM (n = 2 mice, HD 2.2). **B)** Representative immunofluorescence images of human cells (hNuclei$^+$ or STEM121$^+$) expressing S100β (astrocytes), GSTπ (oligodendrocytes), or β-tubulin III (neurons) markers in the cerebellum (CB) or spinal cord (SC). Nuclei were counterstained with Hoechst. Arrows indicate co-localization between immunofluorescence signals. **C)** Bar plot showing the percentage of hiPSC-NSC-derived GSTπ$^+$ (red bars) or S100β$^+$ (blue bars) cells co-expressing SOX10 marker (scattered bars). Percentages were calculated as: (number of STEM121$^+$GSTπ$^+$Sox10$^+$ or STEM121$^+$S100β$^+$Sox10$^+$ cells) / (number of STEM121$^+$GSTπ$^+$ or STEM121$^+$S100β$^+$ cells) × 100. Data are expressed as mean ± SEM (n = 6 mice). **D)** Representative immunofluorescence images of human cells (STEM121$^+$; red) co-expressing SOX10 (green) and GSTπ (blue) or SOX10 (green) and S100β (blue) (arrows). **A-D)** Transplanted hiPSC-NSC clones HD 1.1 (p2), HD 1.3 (p2), and HD 2.2 (p1-3). Source data are provided as a Source Data file.

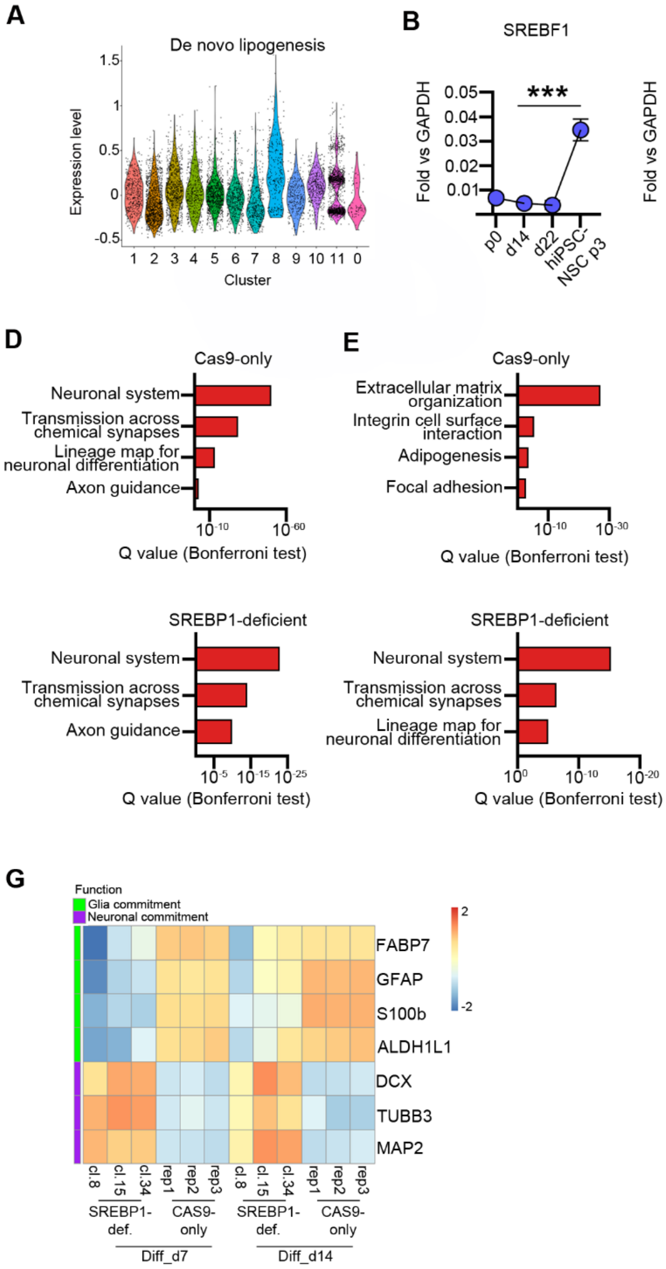

Supplementary Figure 8. Molecular analyses in SREBP-1 deficient cells. A) Seurat violin plots showing the expression levels in scRNA-seq clusters of the gene signature associated with *de novo* lipogenesis. **B)** Time-course qRT-PCR analysis of *SREBF1* expression during hiPSC-to-NSC differentiation. Timepoints analyzed: hiPSCs (day 0), rosette-like formations (day 14), hiPSC-NSC maturation (day 22), and hiPSC-NSCs at passage 3. Expression levels are normalized on the housekeeping gene *GAPDH*. Each dot represents the mean ± SEM of 3 biological replicates/time point. One-way ANOVA followed by Dunn's multiple comparison test: ***, $p < 0.001$. **C)** Bar plot showing representative qRT-PCR data of the expression levels of *SREBF1* mRNA in pooled SREBP1 KO hiPSCs as compared to control cells treated only with Cas9 protein (Cas9 only) and untreated samples (Mock). Expression levels are normalized on the housekeeping gene *GAPDH*. **D)** Pathways upregulated in SREBP1-deficient and Cas9-only hiPSC-NSCs vs. hiPSCs (RNA-seq analyses). **E)** Pathways upregulated in SREBP1-deficient and Cas9-only hiPSC-NSCs vs. day 14 mixed glia/neuron cultures (RNA-seq analyses). **F)** Pathways upregulated (red bars) and downregulated (blue bars) in Cas9-only vs. SREBP1-def day 14 mixed glia/neuron cultures. **G)** Heatmap showing the expression levels in SREBP1-deficient and Cas9-only mixed glia/neuron cultures (day 7 and day 14) of glia and neuronal markers. Color scale indicates the relative fold change of normalized expression levels of these genes in each sample (blue, low; red, high) (RNA-seq analyses). Source data are provided as a Source Data file.

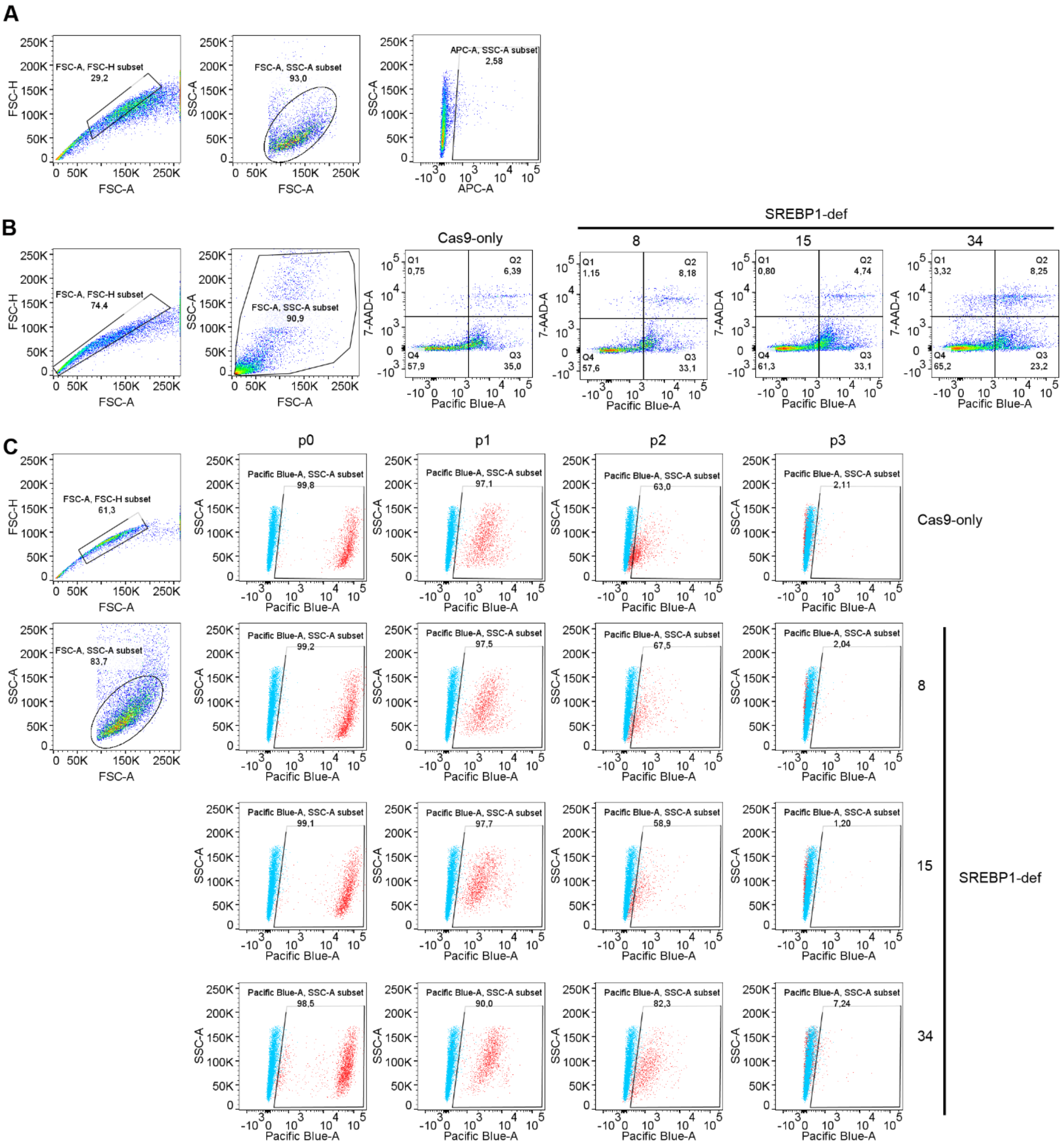

Supplementary Figure 9. Sample gating strategies in flow cytometry analyses. A) Sample gating strategy of plots reported in Supplementary Figure 1C. FSC-A/FSC-H gates were performed to select single cells and exclude doublets. FSC/SCC gates encompassed all live cells in the population to exclude debris. Unstained samples were used as negative controls to define the gate of SSEA4-APC labeled cells (see panel C of Suppl.Fig.1). **B)** Sample gating strategy related to data reported in Figure 7F. FSC-A/FSC-H gates were performed to select single cells and exclude doublets. Cells were stained with Annexin V and 7-AAD. Annexin V^-/7-AAD^- cells were positively selected as living cells, Annexin V^+/7-AAD^- cells as early apoptotic cells, and Annexin V^+/7-AAD^+ cells as late apoptotic cells. Unstained samples were used as negative controls to define the gating strategy. Representative plots of stained Cas9-only and SREBP1-deficient hiPSC-NSCs are reported. **C)** Sample gating strategy related to data reported in Figure 7G. FSC-A/FSC-H gates were performed to select single cells and exclude doublets. FSC/SCC gates encompassed all live cells in the population to exclude debris. hiPSC-NSCs were stained with eBioscience™ Cell Proliferation Dye eFluor™ 450 and analyzed at different cell culture passages. For each analysis, the cytometer was calibrated using rainbow beads. Proliferating hiPSC-NSCs were identified in the SSC/Pacific $Blue^+$ gate. Unstained samples were used

as negative controls to define the gating strategy. Representative plots of Cas9-only and SREBP1-deficient hiPSC-NSCs stained at p0 and analyzed at p0-p3 to evaluate the proliferation rate.

Supplementary Tables

Supplementary Table 1. Summary of hiPSC clones generated by reprogramming of healthy donor (HD) fibroblasts. Skin fibroblasts derived from adult (HD1) and newborn (HD2) healthy donors were transduced with a monocistronic Cre-excisable lentiviral vector (LV) carrying OCT4, SOX2, and KLF4 under the control of the human SFFV promoter, as previously reported[34].

ID	**Fibroblasts** (code number/source)	Age	hiPSC clones
HD1	FFF0561980 / Gaslini Biobank	Adult	HD 1.1
			HD 1.3
HD2	C0045C / Invitrogen	Newborn	HD 2.2
			HD 2.3

Supplementary Table 2. Upstream regulators identified by Ingenuity Pathway Analysis (IPA) on RNA-seq analysis of hiPSC-NPCs vs. hiPSCs. IPA analysis identified the upstream regulators of genes up- or down-regulated during hiPSC neural commitment. Gene name (Upstream Regulator), the inferred activation states of predicted transcriptional regulators (Predicted Activation State), activation *z*-score ($|z\text{-score}| > 2$) and the *p*-value of the overlap (Fisher's Exact Test) between the dataset genes and the genes that are regulated by an Upstream Regulator (*p*-value of overlap) are reported in the table.

Upstream Regulator	Predicted Activation State	Activation *z*-score	*p*-value of overlap
RBL1	Activated	2.556	9.06E-07
FOXO3	Activated	2.514	6.49E-03
MEF2D	Activated	2.480	6.34E-04
NEUROD1	Activated	3.261	5.92E-03
CDKN2A	Activated	3.688	5.54E-07
KLF15	Activated	2.295	4.83E-02
HEY2	Activated	2.006	4.56E-03
SMARCB1	Activated	2.146	3.91E-08
ZBTB17	Activated	2.219	3.90E-03
HDAC1	Activated	3.425	3.25E-04
KDM5B	Activated	3.102	3.05E-06
OTX2	Activated	2.550	2.73E-02
TWIST1	Activated	2.018	2.72E-02
MXD1	Activated	2.213	2.29E-03
CREBBP	Activated	2.339	2.24E-04
EN1	Activated	2.213	2.23E-03
ZEB2	Activated	2.080	2.06E-04
PAX6	Activated	2.837	2.03E-02
ZEB1	Activated	2.042	1.99E-03
KDM5A	Activated	2.194	1.90E-02
NUPR1	Activated	3.580	1.71E-06
HDAC2	Activated	3.115	1.64E-03
SMARCA4	Activated	2.073	1.42E-06
E2F6	Activated	2.688	1.25E-06
FOXN4	Activated	2.236	1.21E-04
TCF3	Activated	2.461	1.07E-02
E2F1	Inhibited	-4.041	1.89E-15
MYCN	Inhibited	-3.976	2.60E-04
POU5F1	Inhibited	-3.589	9.00E-06
CCND1	Inhibited	-2.852	7.74E-15
NANOG	Inhibited	-2.470	9.34E-06
MYCBP	Inhibited	-2.000	1.69E-02

MYC	Inhibited	-6.979	3.59E-10
E2F3	Inhibited	-4.397	7.50E-10
TAL1	Inhibited	-3.471	2.24E-05
SMAD7	Inhibited	-3.415	1.53E-02
MAX	Inhibited	-2.902	1.83E-04
TRIM24	Inhibited	-2.828	2.37E-02
REST	Inhibited	-2.698	2.30E-04
BMI1	Inhibited	-2.607	4.86E-04
EPAS1	Inhibited	-2.408	1.35E-05
ZNF217	Inhibited	-2.385	1.28E-06
POU4F2	Inhibited	-2.359	4.04E-05
E2F2	Inhibited	-2.325	2.89E-09
MNT	Inhibited	-2.236	1.68E-03
SIN3A	Inhibited	-2.236	3.58E-03
ZIC2	Inhibited	-2.219	8.50E-03
HNF1B	Inhibited	-2.205	2.08E-04
MED1	Inhibited	-2.197	1.20E-07
HAND2	Inhibited	-2.139	2.36E-02
TFAP2C	Inhibited	-2.094	6.53E-03
HOXA10	Inhibited	-2.063	3.41E-03
MYCL	Inhibited	-2.000	3.19E-03

Supplementary Table 3. Upstream regulators identified by Ingenuity Pathway Analysis (IPA) on the dataset of genes close to hiPSC- and hiPSC-NSC-specific enhancers. IPA analysis of integrated ChIP-seq and RNA-seq datasets identified the upstream regulators of genes associated with gained and lost enhancers (400 kb window) in hiPSC-NSCs. Gene name (Upstream Regulator), the inferred activation states of predicted transcriptional regulators (Predicted Activation State), activation *z*-score (| *z*-score | > 2) and the *p*-value of the overlap between the dataset genes and the genes that are regulated by an Upstream Regulator (*p*-value of overlap - Fisher's Exact Test) are reported in the table.

**Upstream Regulator**	**Predicted Activation State**	**Activation *z*-score**	***p*-value of overlap**
NONO	Activated	2.711	4.17E-04
SREBF1	Activated	3.215	6.41E-04
STAT1	Activated	3.416	9.59E-04
EHMT1	Activated	3.162	2.16E-02
CTNNB1	Activated	2.425	2.38E-02
RELA	Activated	2.534	3.77E-02
TEAD2	Activated	2.236	4.12E-02
E2F1	Inhibited	-3.022	9.23E-19
ATF3	Inhibited	-3.281	1.54E-10
MYCN	Inhibited	-2.599	2.71E-09
MYC	Inhibited	-4.925	2.37E-08
E2F3	Inhibited	-3.160	3.08E-08
MITF	Inhibited	-4.914	8.61E-08
TP63	Inhibited	-3.261	2.87E-07
YAP1	Inhibited	-3.334	1.77E-06
MYBL2	Inhibited	-2.425	4.49E-05
MED1	Inhibited	-4.069	6.66E-05
PAX8	Inhibited	-2.392	8.77E-05
TFAP2C	Inhibited	-2.178	1.23E-04
HIF1A	Inhibited	-4.095	1.66E-04

MYB	Inhibited	-2.615	2.94E-04
SP1	Inhibited	-2.559	3.51E-04
FOXM1	Inhibited	-3.917	4.40E-04
NCOA3	Inhibited	-3.136	4.61E-04
EPAS1	Inhibited	-2.931	6.68E-04
TCF4	Inhibited	-4.382	8.83E-04
ZNF281	Inhibited	-2.200	1.03E-03
MYCBP	Inhibited	-2.000	1.20E-03
FUS	Inhibited	-2.236	1.26E-03
POU5F1	Inhibited	-2.646	1.26E-03
WWTR1	Inhibited	-2.178	1.44E-03
ID1	Inhibited	-2.425	3.91E-03
RELB	Inhibited	-2.376	4.58E-03
GATA4	Inhibited	-2.476	5.22E-03
CREB1	Inhibited	-2.813	7.60E-03
KDM3A	Inhibited	-2.595	8.50E-03
NFKB1	Inhibited	-3.273	1.06E-02
MYOCD	Inhibited	-2.466	1.31E-02
NANOG	Inhibited	-2.359	1.38E-02
EZH2	Inhibited	-2.335	2.45E-02
SRF	Inhibited	-2.219	2.79E-02
CTNNB1	Inhibited	-2.888	3.02E-02
SMARCA4	Inhibited	-4.526	3.30E-02
POU2F2	Inhibited	-3.162	3.43E-02
STAT3	Inhibited	-3.407	4.86E-02

Supplementary Table 4. Upstream regulators identified by Ingenuity Pathway Analysis (IPA) on RNA-seq analysis of hiPSC-NSCs vs. hfNSCs. IPA analysis identified the upstream regulators of genes upregulated in hiPSC-NSCs (*z*-score > 2) or hfNSCs (*z*-score < 2). Gene name (Upstream Regulator), the inferred activation states of predicted transcriptional regulators (Predicted Activation State), activation *z*-score, and the *p*-value of the overlap between the dataset genes and the genes that are regulated by an Upstream Regulator (*p*-value of overlap, Fisher's Exact Test) are reported in the table.

**Upstream Regulator**	**Predicted Activation State**	**Activation *z*-score**	***p*-value of overlap**
NKX2-3	Activated	4.633	1.33E-11
CTNNB1	Activated	4.249	3.83E-22
NEUROG3	Activated	3.977	2.81E-04
MYC	Activated	3.853	1.14E-08
TRIM24	Activated	3.429	2.66E-05
KLF4	Activated	3.158	3.56E-08
HIF1A	Activated	3.123	1.41E-08
SMAD3	Activated	3.098	3.52E-04
HOXA9	Activated	3.065	3.12E-04
SRF	Activated	2.989	1.51E-05
SOX11	Activated	2.784	4.04E-05
ATF4	Activated	2.692	4.71E-04
KMT2D	Activated	2.571	1.79E-04
HDAC6	Activated	2.547	1.07E-02
NOTCH3	Activated	2.456	1.16E-02
GFI1	Activated	2.397	5.45E-03
HNF1A	Activated	2.380	2.41E-03
LEF1	Activated	2.355	2.50E-03

GLI1	Activated	2.346	3.90E-09
LHX1	Activated	2.340	1.17E-04
STAT3	Activated	2.303	9.38E-09
LMX1B	Activated	2.219	2.85E-03
SIM1	Activated	2.213	1.66E-06
MAML1	Activated	2.173	2.53E-02
SIX5	Activated	2.170	3.60E-04
CDX1	Activated	2.164	4.44E-02
CEBPB	Activated	2.162	4.43E-02
EGR2	Activated	2.158	5.51E-04
SMAD4	Activated	2.101	1.65E-07
ARNT2	Activated	2.101	1.35E-06
FOXO1	Activated	2.030	1.81E-05
OTX2	Activated	2.026	3.38E-04
POU3F2	Activated	2.000	1.79E-02
LMX1A	Activated	2.000	1.27E-02
GLIS1	Activated	2.000	2.35E-03
IRF7	Inhibited	-3.331	3.14E-04
PAX1	Inhibited	-3.207	6.77E-03
TFEB	Inhibited	-3.153	4.73E-04
NLRC5	Inhibited	-3.113	2.77E-04
REST	Inhibited	-3.010	5.73E-18
STAT2	Inhibited	-2.721	1.07E-03
IRF1	Inhibited	-2.658	1.46E-02
PRDM8	Inhibited	-2.646	7.30E-04
SOX3	Inhibited	-2.475	1.87E-09
COMMD3-BMI1	Inhibited	-2.379	1.74E-04
TP53	Inhibited	-2.254	2.19E-16
HOXC9	Inhibited	-2.236	1.79E-02
GATA3	Inhibited	-2.203	2.16E-02
GMNN	Inhibited	-2.191	3.41E-07
SOX1	Inhibited	-2.191	2.04E-08
SPDEF	Inhibited	-2.165	2.14E-07
ZNF217	Inhibited	-2.065	2.31E-07
NFKB1	Inhibited	-2.009	7.44E-03

Supplementary Table 5. List of gRNAs and primers used for PCR amplification and sequencing of Cas9 on-target sequence. Sequencing results showing the Cas9-nuclease edited sequences (*SREBF1* exon 5) in SREBP1-deficient clones.

**gRNA**	
gRNA1	GAGCTCAAGGATCTGGTGGT
gRNA2	TGCGCTTCTCTCCACGGCTC
gRNA3	CGGAGAAGCTGCCTATCAAC
**Sequencing primers**	
FW	5’-TAGCACAGCCCCACCTTTAT-3’
Rev	5’-AGCCATGAAGACAGACGGAG-3’
**Clone**	**Edited on-target sequence (*SREBF1* exon 5)**
8	CCACACCTTTGCCTCAGTGCCCAC|-----C---134bp deletion--|GATAGGCAGCTTCTCCGCATCTACG
15	CCACACCTTTGCCTCAGTGCCCACC|-----C---134bp deletion--|GATAGGCAGCTTCTCCGCATCTACG

34	CCACACCTTTGCCTCAGTGCCCACC|---134bp deletion--|GATAGGCAGCTTCTCCGCATCTACG

Supplementary Table 6. List of primers used for qRT-PCR on immunoprecipitated chromatin.

Primers for ChIP analysis	
**Region**	**Sequence**
Negative	Fw 5' -AAAGCTGGACTGGTGAATGC- 3'
	Rev 5' -TCAAAGGCTCATCTTTGCAG- 3'
FGFR1	Fw 5' -GTCACAGCTGCCATCCTACA- 3'
	Rev 5' -TCTATTTGGGGACTCCGAGA- 3'
LIN28	Fw 5' -CTCAGCAGTGGATGGGGATG- 3'
	Rev 5' -GCAGGAGGAACCCAAAGAGT- 3'
PPAP2B	Fw 5' -TGAGCATCGCTTTTCTGGGG- 3'
	Rev 5' -ACAGCTTGCTACGAGACAGG- 3'

Supplementary Table 7. List of primers and probes used for SYBR Green and TaqMan qRT-PCR.

SYBR Green primers	
**Gene**	**Sequence**
PAX6	Fw 5' -AGTGAATCAGCTCGGTGGTGTCTT- 3'
	Rev 5' -TGCAGAATTCGGGAAATGTCGC- 3'
ROBO2	Fw 5' -TTCTTCTTGCGCATCGTGC- 3'
	Rev 5' -CGCATTTCGACTCACTGCTTC- 3'
OCT4	Fw 5' -TCGAGAACCGAGTGAGAGG- 3'
	Rev 5' -GAACCACACTCGGACCACA- 3'
NANOG	Fw 5' -ATGCCTCACACGGAGACTGT- 3'
	Rev 5' -AAGTGGGTTGTTTGCCTTTG- 3'
LIN28	Fw 5' -GAAGCGCAGATCAAAAGGAG- 3'
	Rev 5' -GCTGATGCTCTGGCAGAAGT- 3'
**TaqMan probes**	
**Gene**	**Code**
PAX6	Hs00240871_m1
NEUROD1	Hs01922995_s1
MXD1	Hs00965581_m1
MAX	Hs00231142_m1
MYC	Hs00153408_m1
SREBF1	Hs01088691_m1
ZEB1	Hs00232783_m1
GAPDH	Hs99999909_m1
NKX2-2	Hs00159616_m1
NR3C2	Hs01031804_m1
GRHL1	Hs01119372_m1
MEIS1	Hs00180020_m1
PPARA	Hs00947536_m1
CREB5	Hs00191719_m1
POU3F2	Hs00271595_s1
ZNF711	Hs00944896_m1
NPAS2	Hs00231212_m1
RXRB	Hs00232774_m1
IRF2	Hs01082884_m1
JUN	Hs01103582_s1
ARNT	Hs01121918_m1
MEF2A	Hs01050406_g1
FOXA1	Hs04187555_m1
NR3C1	Hs00353740_m1
TRIM3	Hs01548703_m1

ARNT2	Hs00977663_m1
HES1	Hs00172878_m1
NUPR1	Hs01044304_g1
FOXN4	Hs01566111_m1
FOXG1	Hs01850784_s1

Supplementary Table 8. List of primary and secondary antibodies with antigen, host species, provider, product number, and working dilutions indicated.

	**Primary Antibodies**	
**Antigen**	**Host species (provider, product number)**	**Working Dilution**
MAP2	Mouse IgG1 (Immunlogical Science, MAB10334)	1:300
GFAP	Mouse monoclonal IgG1 (Millipore, MAB3402)	1:1000
Human Nuclei	Mouse monoclonal (Sigma-Aldrich, MAB1281)	1:100
STEM121	Mouse monoclonal (Takara Bio, Y40410)	1:100
Human Mitochondria	Mouse monoclonal (Millipore, MAB1273)	1:100
GSTπ	Rabbit polyclonal (MBL, 312)	1:500
SOX10	Goat polyclonal IgG (R&D Systems, AF2864)	1:100
β-tubulin III	Rabbit polyclonal IgG (BioLegend, 802001)	1:2000
S100 β	Rabbit (Swant, 37A/ Proteintech, 15146-1-ap)	1:1000
Human Nestin	Rabbit polyclonal (Millipore, ABD69)	1:200
Ki67 (D3B5)	Rabbit monoclonal IgG (Cell Signaling, MAB9129)	1:200
Ki67	Mouse polyclonal (Novocastra, NCL-Ki67-MM1)	1:100
	**Secondary antibodies**	
Alexa 488	Goat anti-Mouse IgG (Mol Probes, A11001)	1:1000
Alexa 488	Donkey anti-Goat IgG (Mol Probes, A11055)	1:1000
Alexa 546	Goat anti-Rabbit IgG (Mol Probes, A11010)	1:2000
Cy3	Donkey anti-Mouse IgG (Millipore, AP192C)	1:1000
Alexa 647	Donkey anti-Rabbit IgG (Invitrogen, A31573)	1:500
	**Nuclear Counterstain**	
Hoechst 33342	(Invitrogen, H3570)	1:1000
