# Supplementary material for: Human iPSC-derived neural stem cells displaying radial glia signature exhibit long-term safety in mice
Source: Nat Commun. 2024 Nov 1;15:9433. doi: 10.1038/s41467-024-53613-7 (PMC11530573; doi:10.1038/s41467-024-53613-7)
Supplement: Supplementary file 2 — Description of Additional Supplementary Files [file 41467_2024_53613_MOESM2_ESM.pdf]

Description of Additional Supplementary Files

File Name: Supplementary Data 1

Description: Analysis of RNA-seq data

File Name: Supplementary Data 2

Description: Analysis of ChIP-seq data

File Name: Supplementary Data 3

Description: Analysis of scRNA-seq data

File Name: Supplementary Data 4

Description: Analysis of RNA-seq data of SREBP1-deficient cells
