# Supplementary material for: Human iPSC-derived neural stem cells displaying radial glia signature exhibit long-term safety in mice
Source: Nat Commun. 2024 Nov 1;15:9433. doi: 10.1038/s41467-024-53613-7 (PMC11530573; doi:10.1038/s41467-024-53613-7)
Supplement: Supplementary file 7 — Reporting Summary [file 41467_2024_53613_MOESM7_ESM.pdf]

nature portfolio

Corresponding author(s): Angela Gritti
Last updated by author(s): 23/09/24

Reporting Summary

Nature Portfolio wishes to improve the reproducibility of the work that we publish. This form provides structure for consistency and transparency in reporting. For further information on Nature Portfolio policies, see our Editorial Policies and the Editorial Policy Checklist.

Statistics

For all statistical analyses, confirm that the following items are present in the figure legend, table legend, main text, or Methods section.

n/a	Confirmed	
☐	☒	The exact sample size (*n*) for each experimental group/condition, given as a discrete number and unit of measurement
☐	☒	A statement on whether measurements were taken from distinct samples or whether the same sample was measured repeatedly
☐	☒	The statistical test(s) used AND whether they are one- or two-sided<br>*Only common tests should be described solely by name; describe more complex techniques in the Methods section.*
☒	☐	A description of all covariates tested
☐	☒	A description of any assumptions or corrections, such as tests of normality and adjustment for multiple comparisons
☐	☒	A full description of the statistical parameters including central tendency (e.g. means) or other basic estimates (e.g. regression coefficient) AND variation (e.g. standard deviation) or associated estimates of uncertainty (e.g. confidence intervals)
☐	☒	For null hypothesis testing, the test statistic (e.g. *F*, *t*, *r*) with confidence intervals, effect sizes, degrees of freedom and *P* value noted<br>*Give P values as exact values whenever suitable.*
☒	☐	For Bayesian analysis, information on the choice of priors and Markov chain Monte Carlo settings
☒	☐	For hierarchical and complex designs, identification of the appropriate level for tests and full reporting of outcomes
☒	☐	Estimates of effect sizes (e.g. Cohen's *d*, Pearson's *r*), indicating how they were calculated

Our web collection on statistics for biologists contains articles on many of the points above.

Software and code

Policy information about availability of computer code

Data collection	FACS data collection: BD FACSDiva Software. Microscopy data collection (Axioscope 5FL, Leica TCS Sp8, and MAVIG RS-G4): LAS X Software (Leica) and RSG4 Software. qRT-PCR data collection: QuantStudio Software (Applied Biosystems).<br>Bulk RNA-seq data: Illumina HiSeq2500 sequencing platform.<br>ChIP-seq data: Illumina HiSeq2500 sequencing platform.<br>scRNA-seq data: High-throughput sequencing was performed on NovaSeq 600 2x150bp paired end (Illumina).
Data analysis	Softwares has been listed in the material & method section of the manuscript.<br>For FACS analysis: FlowJo (version 10.8.1). Microscopy image analysis: ImageJ (Fiji version 2.14.0) and Photoshop CS4. Editing efficiency analysis: Synthego ICE. Statistic analyses: Graphpad 8.0a.<br>Bulk RNA-seq data analysis: FastQC tool, STAR aligner (v2.5.0a), HTSeq (v.0.6.1), DESeq2 R/Bioconductor package (v1.8.1).<br>ChIP-seq data analysis: Bowtie aligner, SAMtools, BEDtools, MACS tool.<br>For single-cell data, CellRanger v6.1.1 software (10X Genomics) was used to perform demultiplexing of the input files, alignment to the human reference genome (GRCh38), and UMI quantification to produce a cell-by-gene matrix for all the samples, which were then imported in the R environment (v.4.1.3) and analysed with Seurat (v4.1.1). In details, cells expressing less than 200 (indication of low-viability) or more than 6000 (indication of doublets) genes, as well as those having more than 20% of transcripts coming from mitochondrial genes (indication of dying cells), were removed. Samples were then merged, and the resulting combined object was analyzed by performing log-normalization with a scale factor of 1000 by using the NormalizedData function of the Seurat package, principal component analysis, batch removal (using Harmony), clustering, and Uniform Manifold Approximation and Projection (UMAP) embedding computation. Cluster markers were computed by using the FindAllMarkers function of the Seurat package, which exploits a Wilcoxon Rank Sum test for significance. The Seurat function AddModuleScore was used to compute the average expression of gene signatures in different set of cells (i.e., samples or clusters), while the R/Bioconductor package clusterProfiler (v4.7.1) was employed to perform enrichment of cluster markers on the KEGG database. Single-cell

pseudotime trajectories were computed with the R package Slingshot (v2.2.1) which exploits previously computed cell clusters, and focusing on the transcriptional lineage that describes the progressive transition from cluster 1 to cluster 7 by also identifying genes whose expression changes along that trajectory.

For manuscripts utilizing custom algorithms or software that are central to the research but not yet described in published literature, software must be made available to editors and reviewers. We strongly encourage code deposition in a community repository (e.g. GitHub). See the Nature Portfolio guidelines for submitting code & software for further information.

Data

Policy information about availability of data

All manuscripts must include a data availability statement. This statement should provide the following information, where applicable:

- Accession codes, unique identifiers, or web links for publicly available datasets
- A description of any restrictions on data availability
- For clinical datasets or third party data, please ensure that the statement adheres to our policy

Bulk RNA-seq and ChIP-seq data have been deposited at GEO under accession number GSE239446.
Single cell RNA-seq data have been deposited at GEO under accession number GSE238206.
Analyzed RNA-seq, ChIP-seq and scRNA-seq data (list of DEGs and GO terms) are available in Suppl. Files 1-4.

Research involving human participants, their data, or biological material

Policy information about studies with human participants or human data. See also policy information about sex, gender (identity/presentation), and sexual orientation and race, ethnicity and racism.

Reporting on sex and gender	*Use the terms sex (biological attribute) and gender (shaped by social and cultural circumstances) carefully in order to avoid confusing both terms. Indicate if findings apply to only one sex or gender; describe whether sex and gender were considered in study design; whether sex and/or gender was determined based on self-reporting or assigned and methods used. Provide in the source data disaggregated sex and gender data, where this information has been collected, and if consent has been obtained for sharing of individual-level data; provide overall numbers in this Reporting Summary. Please state if this information has not been collected. Report sex- and gender-based analyses where performed, justify reasons for lack of sex- and gender-based analysis.*
Reporting on race, ethnicity, or other socially relevant groupings	*Please specify the socially constructed or socially relevant categorization variable(s) used in your manuscript and explain why they were used. Please note that such variables should not be used as proxies for other socially constructed/relevant variables (for example, race or ethnicity should not be used as a proxy for socioeconomic status). Provide clear definitions of the relevant terms used, how they were provided (by the participants/respondents, the researchers, or third parties), and the method(s) used to classify people into the different categories (e.g. self-report, census or administrative data, social media data, etc.) Please provide details about how you controlled for confounding variables in your analyses.*
Population characteristics	*Describe the covariate-relevant population characteristics of the human research participants (e.g. age, genotypic information, past and current diagnosis and treatment categories). If you filled out the behavioural & social sciences study design questions and have nothing to add here, write "See above."*
Recruitment	*Describe how participants were recruited. Outline any potential self-selection bias or other biases that may be present and how these are likely to impact results.*
Ethics oversight	*Identify the organization(s) that approved the study protocol.*

Note that full information on the approval of the study protocol must also be provided in the manuscript.

Field-specific reporting

Please select the one below that is the best fit for your research. If you are not sure, read the appropriate sections before making your selection.

☒ Life sciences ☐ Behavioural & social sciences ☐ Ecological, evolutionary & environmental sciences

For a reference copy of the document with all sections, see nature.com/documents/nr-reporting-summary-flat.pdf

Life sciences study design

All studies must disclose on these points even when the disclosure is negative.

Sample size	For in vitro and bioinformatic analyses, sample sizes were chosen (n=3-4 hiPSC clones) to exclude effects associated to the reprogramming and differentiation processes. For in vivo analyses, hiPSC-derived NSCs were transplanted in a number of mice sufficient to exclude variability associated with transplantation procedures (n=2-6 mice/group). For each experiment, the sample size is detailed in the figure legend. No methods were used to predetermine sample size based on analyses from previous published work (https://doi.org/10.5966/sctm.2015-0414) which showed that data collected in selected hiPSC-NSC lines and parental hiPSCs were reproducible and 3-4 samples captured variation.

Data exclusions	No data were excluded from the analyses
Replication	Where indicated in the paper, experiments were replicated in multiple biological replicates and differentiation batches. For bulk RNA-seq, ChIP-seq and scRNA-seq analyses each experiment was performed in 3-4 biological replicates by using different differentiation batches.
Randomization	hiPSC donors and clones were not randomized for bulk RNA-seq, ChIP-seq and scRNA-seq analyses because no experimental groups were defined in these studies. In SREBF1-deficient experiments, samples were not randomized to experimental groups because treatment groups (SREBF1-deficient clones) were paired with their isogenic controls from the same differentiation batch. Neonatal animals were randomly selected for intracerebral injections in the in vivo study.
Blinding	Investigators were not blinded since the same parameters were applied to all samples without adjustments.

Behavioural & social sciences study design

All studies must disclose on these points even when the disclosure is negative.

Study description	*Briefly describe the study type including whether data are quantitative, qualitative, or mixed-methods (e.g. qualitative cross-sectional, quantitative experimental, mixed-methods case study).*
Research sample	*State the research sample (e.g. Harvard university undergraduates, villagers in rural India) and provide relevant demographic information (e.g. age, sex) and indicate whether the sample is representative. Provide a rationale for the study sample chosen. For studies involving existing datasets, please describe the dataset and source.*
Sampling strategy	*Describe the sampling procedure (e.g. random, snowball, stratified, convenience). Describe the statistical methods that were used to predetermine sample size OR if no sample-size calculation was performed, describe how sample sizes were chosen and provide a rationale for why these sample sizes are sufficient. For qualitative data, please indicate whether data saturation was considered, and what criteria were used to decide that no further sampling was needed.*
Data collection	*Provide details about the data collection procedure, including the instruments or devices used to record the data (e.g. pen and paper, computer, eye tracker, video or audio equipment) whether anyone was present besides the participant(s) and the researcher, and whether the researcher was blind to experimental condition and/or the study hypothesis during data collection.*
Timing	*Indicate the start and stop dates of data collection. If there is a gap between collection periods, state the dates for each sample cohort.*
Data exclusions	*If no data were excluded from the analyses, state so OR if data were excluded, provide the exact number of exclusions and the rationale behind them, indicating whether exclusion criteria were pre-established.*
Non-participation	*State how many participants dropped out/declined participation and the reason(s) given OR provide response rate OR state that no participants dropped out/declined participation.*
Randomization	*If participants were not allocated into experimental groups, state so OR describe how participants were allocated to groups, and if allocation was not random, describe how covariates were controlled.*

Ecological, evolutionary & environmental sciences study design

All studies must disclose on these points even when the disclosure is negative.

Study description	*Briefly describe the study. For quantitative data include treatment factors and interactions, design structure (e.g. factorial, nested, hierarchical), nature and number of experimental units and replicates.*
Research sample	*Describe the research sample (e.g. a group of tagged Passer domesticus, all Stenocereus thurberi within Organ Pipe Cactus National Monument), and provide a rationale for the sample choice. When relevant, describe the organism taxa, source, sex, age range and any manipulations. State what population the sample is meant to represent when applicable. For studies involving existing datasets, describe the data and its source.*
Sampling strategy	*Note the sampling procedure. Describe the statistical methods that were used to predetermine sample size OR if no sample-size calculation was performed, describe how sample sizes were chosen and provide a rationale for why these sample sizes are sufficient.*
Data collection	*Describe the data collection procedure, including who recorded the data and how.*
Timing and spatial scale	*Indicate the start and stop dates of data collection, noting the frequency and periodicity of sampling and providing a rationale for these choices. If there is a gap between collection periods, state the dates for each sample cohort. Specify the spatial scale from which the data are taken*
Data exclusions	*If no data were excluded from the analyses, state so OR if data were excluded, describe the exclusions and the rationale behind them, indicating whether exclusion criteria were pre-established.*

Reproducibility	*Describe the measures taken to verify the reproducibility of experimental findings. For each experiment, note whether any attempts to repeat the experiment failed OR state that all attempts to repeat the experiment were successful.*
Randomization	*Describe how samples/organisms/participants were allocated into groups. If allocation was not random, describe how covariates were controlled. If this is not relevant to your study, explain why.*
Blinding	*Describe the extent of blinding used during data acquisition and analysis. If blinding was not possible, describe why OR explain why blinding was not relevant to your study.*

Did the study involve field work? ☐ Yes ☐ No

Field work, collection and transport

Field conditions	*Describe the study conditions for field work, providing relevant parameters (e.g. temperature, rainfall).*
Location	*State the location of the sampling or experiment, providing relevant parameters (e.g. latitude and longitude, elevation, water depth).*
Access & import/export	*Describe the efforts you have made to access habitats and to collect and import/export your samples in a responsible manner and in compliance with local, national and international laws, noting any permits that were obtained (give the name of the issuing authority, the date of issue, and any identifying information).*
Disturbance	*Describe any disturbance caused by the study and how it was minimized.*

Reporting for specific materials, systems and methods

We require information from authors about some types of materials, experimental systems and methods used in many studies. Here, indicate whether each material, system or method listed is relevant to your study. If you are not sure if a list item applies to your research, read the appropriate section before selecting a response.

Materials & experimental systems

n/a	Involved in the study	
☐	☒	Antibodies
☐	☒	Eukaryotic cell lines
☒	☐	Palaeontology and archaeology
☐	☒	Animals and other organisms
☒	☐	Clinical data
☒	☐	Dual use research of concern
☒	☐	Plants

Methods

n/a	Involved in the study	
☐	☒	ChIP-seq
☐	☒	Flow cytometry
☒	☐	MRI-based neuroimaging

Antibodies

Antibodies used

Primary antibodies: anti-Human Nuclei (1:100, Sigma-Aldrich, MAB1281, clone 235-1, lot. 3543073, 3756536, 3065575), STEM121 (1:100, Takara Bio, Y40410, Lot. AKZ0006S), MAP2 (1:300, Immunlogical Science, MAB10334, Lot. S0040606), GFAP (1:1000 Millipore, MAB3402, Lot.3843675), GSTπ (1:500, MBL, 312, Lot. 063), SOX10 (1:100, R&D Systems, AF2864, Lot. VRY1221031), β-tubulin III (1:2000, BioLegend, 802001, Clone Poly18020, Lot. B324313), S100 β (1:1000, Swant, 37A; 1:500 Proteintech, 15146-1-ap, Lot. 00120806), Human Nestin (1:200, Millipore, ABD69, Lot. 3537114) Ki67 (D3B5), (1:200, Cell Signaling, MAB9129, Lot. 3), Ki67 (1:100, Novocastra, NCL-Ki67-MM1, lot. 6062741), anti-SREBP1 (1:100 MABS1987, Sigma-Aldrich Lot. 3111491).

Secondary antibodies: Alexa 488 Goat anti-Mouse IgG (1:1000, Mol Probes, A11001), Alexa 546 Goat anti-Rabbit IgG (1:2000, Mol Probes, A11010), Alexa 488 Donkey anti-Goat IgG (1:1000, Mol Probes, A11055), Cy3 Donkey anti-Mouse IgG (1:1000, Millipore, AP192C), Alexa 647 Donkey anti-Rabbit IgG (1:500, Invitrogen, A3157),HRP-conjugated goat anti-rabbit (1:10,000 Chemicon, AP132P)

Nuclear Counterstain: Hoechst 33342 (1:1000, Invitrogen, H3570)

Validation

Mouse anti-human nuclei (Sigma-Aldrich, MAB1281), rabbit anti-GSTπ (MBL, 312), Rabbit anti-S100 β (Swant, 37A), Mouse anti-Ki67 (Novocastra, NCL-Ki67-MM1), mouse anti-MAP2 (Immunlogical Science, MAB10334), mouse anti-GFAP (Millipore, MAB3402), and Rabbit anti-β-tubulin III (BioLegend, 802001, previously PRB-435P) were used in previous papers (https://doi.org/10.5966/sctm.2015-0414; https://doi.org/10.1038/s41419-018-0737-0; https://doi.org/10.1016/bs.mcb.2022.04.007).
According to the manufacturer's website:
Mouse STEM121 (Takara Bio, Y40410,) has been validated for immunofluorescence to detect transplanted human neural stem cells in the mouse brain in Salazar DL et al PlosOne 2010.
Goat anti-SOX10 (R&D Systems, AF2864) has been cited in 48 publications, and was validated in BG01V Human Embryonic Stem Cells.
Rabbit anti-Human Nestin (Millipore, ABD69) has been validated in human U251 glioblastoma cells.

Rabbit anti-S100 β (Proteintech, 15146-ap) has been cited in 97 publications, and was validated in human astrocytes, A375 cells.
Rabbit anti-Ki67 (D3B5) (Cell Signaling, MAB9129) has been cited in 342 publications, and was validated for immunofluorescence in ventricular zone in P21 mouse brain.
Rabbit anti-SREBP1(Sigma-Aldrich, MABS1987) has been validated for Western Blotting in HepG2 cell lysates.

Eukaryotic cell lines

Policy information about cell lines and Sex and Gender in Research

Cell line source(s)	The hiPSC clones were previously generated through reprograming of fibroblasts obtained from the Cell Line and DNA Bank of Patients affected by Genetic Diseases (Institute Gaslini, Genova, Italy, http://www.gaslini.org) for HD1 or purchased from Invitrogen (C0045C/ Invitrogen) for HD2.<br><br>hfNSCs were purified from the diencephalic/telencephalic brain region of human (h) fetuses at 10.5-week gestational age as previously described (https://doi.org/10.1371/journal.pone.0010145).
Authentication	HD1 and HD2 hiPSC clones were characterized in Q&C analyses as reported in https://doi.org/10.5966/sctm.2015-0414. hfNSCs were in vitro characterized as reported in https://doi.org/10.1371/journal.pone.0010145.
Mycoplasma contamination	All cell lines were negative for mycoplasma contaminations as evaluated by PCR assays and were cultured in mycoplasma-free environment.
Commonly misidentified lines (See ICLAC register)	No commonly misidentified lines were used in this study

Animals and other research organisms

Policy information about studies involving animals; ARRIVE guidelines recommended for reporting animal research, and Sex and Gender in Research

Laboratory animals	Mus Musculus; Rag-/- gamma-chain-/- C57BL/6 strain; mice were treated at post-natal day 2-4 and analyzed 1.5, 3, and 10 months after treatments
Wild animals	The study did not involved wild animals
Reporting on sex	Sex-based analyses were not performed because gender is not expected to influence the engraftment and behaviour of transplanted hiPSC-NSCs
Field-collected samples	The study did not involve samples collected from the field
Ethics oversight	All experiments and procedures described in this study were performed according to protocols 690 approved by the internal Institutional Animal Care and Use Committee and reported to the Italian Ministry of 691 Health, as required by Italian law (IACUC # 931, #1039).

Note that full information on the approval of the study protocol must also be provided in the manuscript.

Plants

Seed stocks	*Report on the source of all seed stocks or other plant material used. If applicable, state the seed stock centre and catalogue number. If plant specimens were collected from the field, describe the collection location, date and sampling procedures.*
Novel plant genotypes	*Describe the methods by which all novel plant genotypes were produced. This includes those generated by transgenic approaches, gene editing, chemical/radiation-based mutagenesis and hybridization. For transgenic lines, describe the transformation method, the number of independent lines analyzed and the generation upon which experiments were performed. For gene-edited lines, describe the editor used, the endogenous sequence targeted for editing, the targeting guide RNA sequence (if applicable) and how the editor was applied.*
Authentication	*Describe any authentication procedures for each seed stock used or novel genotype generated. Describe any experiments used to assess the effect of a mutation and, where applicable, how potential secondary effects (e.g. second site T-DNA insertions, mosiacism, off-target gene editing) were examined.*

ChIP-seq

Data deposition

☒ Confirm that both raw and final processed data have been deposited in a public database such as GEO.

☒ Confirm that you have deposited or provided access to graph files (e.g. BED files) for the called peaks.

Data access links<br>*May remain private before publication.*	GEO enter token yfobawwctzyjjgl

Files in database submission

For each sample, we deposited raw files (ip-hf-ac_TGACCA_L001_R1_001.fastq.gz, LF_IP_02_trimmed_scaled.fastq.gz, LF_IP_1B_trimmed_scaled.fastq.gz, input-hf_TTAGGC_L001_R1_001.fastq.gz, ipsc-ac-1_ATCACG_L001_R1_001.fastq.gz, iPSC_H3K27ac_WTsan5.fastq.gz, ipsc-ac-2_CGATGT_L001_R1_001.fastq.gz, iPSC_H3K27ac_nWT5.fastq.gz, iPSC_input_WTsan5.fastq.gz, hinsc-ac-1_ACAGTG_L001_R1_001.fastq.gz, hiNSC_H3K27ac_WTsan5.fastq.gz, hinsc-ac-2_GCCAAT_L002_R1_001.fastq.gz, hiNSC_H3K27ac_nWT5.fastq.gz, hiNSC_input_WTsan5.fastq.gz) and processed files (hfNSC_H3K27ac_8759.macs_FE.bw, hfNSC_H3K27ac_02.macs_FE.bw, hfNSC_H3K27ac_1B.macs_FE.bw, iPSC_H3K27ac_WTsan2.macs_FE.bw, iPSC_H3K27ac_WTsan5.macs_FE.bw, iPSC_H3K27ac_nWT2.macs_FE.bw, iPSC_H3K27ac_nWT5.macs_FE.bw, hiNSC_H3K27ac_WTsan2.macs_FE.bw, hiNSC_H3K27ac_WTsan5.macs_FE.bw, hiNSC_H3K27ac_nWT2.macs_FE.bw, hiNSC_H3K27ac_nWT5.macs_FE.bw).

Genome browser session (e.g. UCSC)

GEO database: accession number GSE239446

Methodology

Replicates

n=4 biological replicates for hiPSCs; n=4 biological replicates for hiPSC-derived NSC; n=3 technical replicates for fetal hNSCs

Sequencing depth

Each library was sequenced in one lane of a single strand 50 bp Illumina HiSeq 2500 run.

Antibodies

Rabbit antibody against H3K27ac (ab4729, Abcam)

Peak calling parameters

ChIP-seq peak calling was performed with MACS2 (with –broad and --qvalue 0.05 options) and using each INPUT data to model the background noise.

Data quality

Quality of each sequenced sample was checked using cross-correlation analysis implemented in spp R package.

Software

Bowtie aligner, SAMtools, BEDtools, MACS tool.

Flow Cytometry

Plots

Confirm that:

☐ The axis labels state the marker and fluorochrome used (e.g. CD4-FITC).

☒ The axis scales are clearly visible. Include numbers along axes only for bottom left plot of group (a 'group' is an analysis of identical markers).

☐ All plots are contour plots with outliers or pseudocolor plots.

☐ A numerical value for number of cells or percentage (with statistics) is provided.

Methodology

Sample preparation

hiPSCs and hiPSC-NSCs were detached with Accutase, whereas hfNSC neurospheres were mechanically disaggregated to single-cell suspensions by pipetting. After washing in PBS, cells were incubated for 30 minutes in PBS+2% FBS with anti-SSEA4 APC-681 conjugated antibody (FAB1435a, R&D Systems). After staining, cells were washed in PBS+2% FBS and collected for FACS Analyses.
For Annexin V/7AAD analysis, we used Dead Cell Apoptosis Kits with Annexin V for Flow Cytometry (Thermo Fisher) according to manufacturer instructions. Cell proliferation assay was performed using eBioscience™ Cell Proliferation Dye eFluor™ 450 (ThermoFisher) according to the manufacturer's instructions. For each day of analysis, the cytometer was calibrated using rainbow beads (Spherotech).

Instrument

BD FACSCanto II (Becton Dickinson)

Software

BD FACSDiva Software and FlowJo software version 10.8.1

Cell population abundance

Cells were not sorted in this study. We analysed a starting population of at least 10,000 cells in each sample.

Gating strategy

Preliminary FSC/SCC gates encompassed all live cells in the population to exclude debris. FSC-A/FSC-H gates were performed to select single cells and exclude doublets. Unstained samples were used as negative control to define the gating strategies. In Figure Supp 1C, cells were gated on SSC/SSEA4-APC. In Figure 7F, cells were stained with Annexin V and 7-AAD. Annexin V-/7-AAD- cells were positively selected as living cells, Annexin V+/7-AAD- cells as early apoptotic , and Annexin V+/7-AAD+ cells as late apoptotic cells. In Figure 7G cells were stained with eBioscience™ Cell Proliferation Dye eFluor™ 450 and gated at different passages on SSC/Pacific Blue. For each analysis the cytometer was calibrated using rainbow beads.

☐ Tick this box to confirm that a figure exemplifying the gating strategy is provided in the Supplementary Information.
